# Analysis of barriers and facilitators to patient activation among patients with infectious keratitis from a social ecosystems theory perspective: a qualitative study in the Chinese context

**DOI:** 10.3389/fpubh.2026.1881288

**Published:** 2026-10-07

**Authors:** Huanle Yu, Jiating Wang, Rui Zhou, Xiao Wang, Yuyang Li, Xintian Wang, Jing Wang, Ziling Song, Xi Zhang, Xiaoqiong Huang

**Affiliations:** 1 School of Ophthalmology and Optometry, Wenzhou Medical University, Wenzhou, Zhejiang, China; 2 School of Nursing, Wenzhou Medical University, Wenzhou, Zhejiang, China; 3 School of Medical Humanities and Management, Wenzhou Medical University, Wenzhou, Zhejiang, China; 4 National Clinical Research Center for Ocular Diseases, Eye Hospital, Wenzhou Medical University, Wenzhou, Zhejiang, China

**Keywords:** keratitis, patient activation, patient participation, qualitative research, social ecosystems theory

## Abstract

**Background:**

Infectious keratitis is one of the leading causes of visual impairment and blindness, placing a significant burden on global public health. Its treatment and favorable prognosis depend heavily on patients' knowledge, skills, and confidence in managing their own health. Nevertheless, qualitative investigations into patient activation among individuals with infectious keratitis within the Chinese cultural context remain scarce. Additionally, research examining this issue through the lens of social ecosystem theory is notably lacking.

**Objective:**

This study aims to identify the facilitators and barriers to patient activation among patients with infectious keratitis in China using a qualitative approach to provide a theoretical basis for developing systematic, multilevel intervention strategies.

**Methods:**

This study employed a descriptive qualitative research design. Using purposive sampling, 15 patients with infectious keratitis were recruited from a tertiary eye hospital in Zhejiang Province, China, between July and September 2025. Semi-structured interviews were conducted, and the interview data were analyzed using qualitative content analysis with NVivo 12.

**Results:**

Based on social ecosystems theory, the analysis identified three main categories at the micro, meso, and macro levels: (1) microsystem —perceptions and behavioral adaptation challenges under disease impact; (2) mesosystem—support and tension in patient interactions with family and providers; (3) macrosystem—challenges and needs in building social networks.

**Conclusions:**

Patient activation in infectious keratitis is constrained by individual, family, healthcare, and social factors across the micro, meso, and macro levels. Future interventions need to incorporate both facilitators and barriers by strengthening patient education, family support, patient-provider communication, and coordination of social resources to enhance patient activation and improve clinical outcomes.

## 1 Introduction

Infectious keratitis is a leading cause of visual impairment and blindness worldwide (1). A global meta-analysis showed that corneal opacity (primarily caused by infectious keratitis) results in approximately 5.467 million people aged 40 and older worldwide being blind in both eyes or experiencing moderate-to-severe visual impairment, with an additional 6.2 million people blind in one eye (2). This issue is particularly severe in developing countries, where it is often referred to as a “silent epidemic” (3) and places a significant burden on both global public health and socioeconomic systems (4). A study by the IRIS Registry indicates that infectious keratitis may account for 6.5% of all ophthalmic visits in the United States (5). In China, surveys indicate that infectious keratitis is the primary cause of corneal blindness, accounting for approximately one-quarter of all blindness cases in the country (6). Infectious keratitis is a corneal infection caused by various microorganisms, including bacteria, fungi, viruses, and parasites (7), also known as infectious corneal ulceration (8). This condition may present with ocular pain, redness, photophobia, and varying degrees of vision loss, accompanied by corneal ulceration (4, 9). If not promptly controlled, infectious keratitis can lead to irreversible corneal damage, manifesting as progressive corneal opacity, perforation, and scar formation (10), ultimately resulting in severe visual impairment or blindness (11).

Due to the prolonged treatment duration, high medication frequency, and recurrent nature of infectious keratitis (12), continuous monitoring and care are required (13, 14). Treatment outcomes and prognosis depend to a large extent on patients' strict, long-term, and proactive adherence, which places high demands on their self-health management capabilities. Good treatment adherence helps control the disease, reduce complications, and improve patients' quality of life (15). However, patients with infectious keratitis (IK) commonly report low treatment adherence (16, 17), primarily manifested by adverse behaviors such as self-discontinuation of medication and delayed follow-up visits. Improving treatment adherence often requires patient activation. As a core element of disease management, patient activation is defined as a patient's knowledge, skills, and confidence in managing their own health—a behavioral concept (18). The level of patient activation significantly influences treatment adherence (19). Patients with low activation often lack disease-related knowledge, health literacy, self-efficacy, or sufficient social support (20), making it difficult for them to effectively manage their condition, which may lead to poor disease control, worsening corneal damage, and a poorer prognosis (21). In contrast, activated patients possess the ability to self-manage and believe they play a crucial role in their disease management (20, 22). A study conducted by Özkan et al. (23) demonstrated that, among individuals with visual impairments, higher patient activation can lead to effective disease management, thereby improving health outcomes and quality of life.

Currently, research on infectious keratitis primarily focuses on clinical disease prevention, diagnosis, and treatment (24, 25). Some scholars have conducted qualitative explorations of the symptom experiences and management strategies of patients with infectious corneal ulcers, revealing a variety of symptom management strategies (26). In addition, a study using a mixed-methods approach investigated healthcare-seeking behaviors in this population and found that patients commonly experienced insufficient disease knowledge and delayed seeking of medical care (27). However, research exploring patient activation in patients with infectious keratitis remains limited. Patient activation, as a core element of disease management, is typically assessed using the Patient Activation Measure (PAM) developed by Hibbard et al. (28), which evaluates patients' activation levels in terms of knowledge, skills, and confidence. Although tools such as the PAM are currently available to quantify patient activation levels, the factors promoting and hindering activation remain unclear among patients with infectious keratitis—a population characterized by visual impairment, complex medication regimens, and cultural diversity—and relevant empirical research remains limited. Furthermore, while quantitative research designs can standardize measurement, they struggle to capture the complexity and situational dependence of individual experiences and cannot deeply reveal patients' authentic experiences (29). Therefore, this study employs qualitative research methods to explore the factors that promote and hinder patient activation from the patients' own perspectives.

It is worth noting that patient activation is determined not only by individual characteristics but is also influenced by family, healthcare providers, and the broader sociocultural and environmental context (30–32). Therefore, identifying the multifaceted factors that promote or hinder patient activation is crucial for developing comprehensive and context-sensitive intervention strategies. Zastrow's social ecosystem theory provides a valuable theoretical framework for exploring this issue. Unlike theories focusing primarily on individual cognitive or behavioral factors, this theory emphasizes the interaction between individuals and their surrounding environments. It divides the ecosystems into the microsystem level (including physiological, psychological, and social subsystems affecting individuals), the mesosystem level (including small-scale groups such as families and occupational groups), and the macrosystem level (including larger systems and groups such as organizations, institutions, policies, and sociocultural factors) (33, 34). The micro-, meso-, and macro-levels were selected as the analytical framework for this study because they provide a coherent structure for interpreting factors at different ecological levels that may influence patient activation. Although previous research has explored the barriers and facilitators of patient activation among older adults with cancer within the social ecosystems framework, that study focused on the long-term management of systemic chronic diseases (35). In contrast, infectious keratitis is an infectious condition characterized by acute onset, rapid progression, intensive treatment regimens (e.g., frequent topical medication and regular follow-up), and visual impairment (12, 36). In addition, the social stigma associated with the visibility of the disease and the overall treatment burden pose unique challenges to patient activation that warrant further investigation. Therefore, guided by the social ecosystems theory and employing a qualitative research design, this study aims to systematically identify the barriers and facilitators of patient activation among individuals with infectious keratitis, examine the interactions across the micro, meso, and macro levels, and delineate the disease-specific characteristics of patient activation in this population, so as to provide both theoretical foundations and practical guidance for the development of more targeted interventions to enhance patient activation in the future.

## 2 Methods

### 2.1 Research design

This study is part of a larger research project aimed at developing a patient-activated intervention program for patients with infectious keratitis. This study employed a qualitative research design with a descriptive approach using content analysis. Face-to-face semi-structured interviews were conducted with 15 Chinese patients with infectious keratitis to explore the facilitating factors and barriers to patient activation. The study was conducted at a tertiary ophthalmology hospital in Zhejiang Province, which is one of the largest specialized ophthalmology centers in the region, offering a wide range of services and serving a diverse patient population. Conducting the study at a single site facilitated in-depth and detailed data collection despite limited resources and ensured consistency in the interview protocol and data collection process. The study adhered to the Consolidated Criteria for Reporting Qualitative Studies (COREQ) (37).

### 2.2 Participants and sampling

Between July and September 2025, this study employed purposive sampling combined with a maximum diversity strategy, focusing on demographic diversity, including patients' gender, age, occupation, disease type, and duration of illness. Disease duration was categorized using a three-month cut-off to distinguish between acute and chronic courses of infectious keratitis (38). Patients with infectious keratitis were recruited from a tertiary ophthalmology hospital in Zhejiang Province, China. The first participant was identified in consultation with the ward nurse manager; the researchers and the ward nurse manager reviewed patient medical records to screen for eligible participants. Subsequent participants were recruited sequentially using the same targeted sampling method to ensure diversity in demographic and clinical characteristics. The sample size was determined by thematic saturation, which is reached when no new themes or ideas emerge after two consecutive interviews and data exhibit information redundancy (39, 40). In this study, the coders observed that no new codes or themes emerged after the 12th interview. Three additional interviews were conducted to confirm saturation stability, and no new themes emerged. Ultimately, a total of 15 participants were recruited. All patients who were approached and met the inclusion criteria agreed to participate. No eligible individuals declined participation or withdrew after recruitment.

Inclusion criteria were as follows: (a) diagnosed with infectious keratitis; (b) age ≥18 years; (c) willing to share their disease experiences; (d) not participating in other studies during this research period. Exclusion criteria were: (a) cognitive or communication impairments that may render them incapable of participating in the study (41); (b) severe psychiatric or neurological disorders that may render them incapable of providing informed consent or participating in the study (42); (c) history of other ocular diseases or vision-affecting surgeries; (d) severe systemic diseases or chronic conditions (such as malignant tumors).

### 2.3 Data collection

This study conducted face-to-face, semi-structured, in-depth interviews with patients meeting the inclusion and exclusion criteria. All interviews were conducted by the first and second authors, both of whom are women pursuing graduate degrees in nursing with a focus on ophthalmic nursing research, and are members of the research team at the hospital where this study was conducted. Prior to data collection, both authors received systematic training in qualitative research as part of their graduate coursework, which included formal instruction and practical training in conducting qualitative interviews. In addition, both authors had participated in other qualitative research projects within the team, gaining practical experience in semi-structured interviewing. The first interview was conducted under the direct supervision of the corresponding author, a university professor with extensive experience in qualitative research. Following the first interview, the corresponding author provided immediate feedback and guidance on interviewing techniques, questioning methods, and follow-up strategies. Subsequent interviews were conducted independently by the two interviewers, with regular team meetings to discuss emerging issues and ensure the quality and consistency of the interviews.

Each interview began with general, open-ended questions (“Could you please share your experiences of managing your eye condition?”), allowing participants to freely describe their experiences before moving on to more specific topics outlined in the interview guide. The interview guide was developed based on social ecosystems theory and a literature review (22, 34, 43, 44). We consulted with experts involved in corneal disease care and selected two patients with infectious keratitis for pre-interviews. The interview guide was modified, and the final version of the interview guide was determined after a group discussion, as shown in Table 1. During the interviews, the interviewer maintained a neutral and non-judgmental stance, avoiding leading questions or personal opinions. Follow-up questions were posed in a non-directive manner to encourage participants to fully elaborate on their responses. All participants were hospitalized for the treatment of infectious keratitis. To minimize the impact of treatment on the study's progress, each interview was limited to 20–50 min and was scheduled during periods when the patient did not require examinations or treatment. Interviews were conducted in a quiet, private interview room, which was coordinated and arranged for this study by the ward nursing staff; only the interviewer and the participant were present during the interview. Each participant was interviewed only once; no repeat interviews were conducted.

**Table 1:** Semi-structured interview guide.

**No**.	**Question**
1	Could you share your psychological experiences during the treatment and management of your illness?
2	Through which channels do you express your requests or needs?
3	How has the illness affected your daily life, work, and family?
4	What are the ways you learn about disease knowledge and disease management skills?
5	What challenges or needs do you encounter in the process of disease treatment and disease management?
6	What factors influence your approach to addressing or resolving these issues?
7	What additional information, assistance, or support would you like to receive?

The interviewer and the participant had no prior relationship. Before the interview began, the researcher established a rapport with the patient through informal conversation, introduced their professional background and motivation for the study, and briefly explained the study's objectives to help the patient relax and feel at ease. This informal conversation was not part of the formal interview. Subsequently, the researchers explained to the patients the purpose, methodology, duration, and confidentiality principles of the interview and informed them that the interview would be audio-recorded. The researchers clarified that the recording would be used solely for transcription purposes, would not be disclosed to any third party, and that all data would be kept strictly confidential and stored securely. Participants' personal information would be anonymized in all transcripts, and participants could withdraw from the study at any time without any consequences. After obtaining the patient's consent and having them sign the informed consent form, the interview was conducted and recorded. During the interview, the researchers observed and recorded the patient's facial expressions and body language while speaking. At the end of each interview, participants had the opportunity to ask questions or share any additional thoughts they considered important. Participants were informed that no follow-up interviews were planned. Participants were invited to review their interview transcripts and the preliminary analysis results. The participants did not suggest any substantive changes, and after discussing the feedback, the research team ultimately decided not to adjust the analysis results.

### 2.4 Data analysis

Data collection and analysis were conducted concurrently in this study. Following each interview, the interviewer transcribed the audio recordings within 24 h and imported the transcripts into Nvivo 12 for data management and analysis. The researchers created a unique file for each participant and used codes ranging from A1 to A15 in place of their real names. Following the qualitative content analysis method proposed by Elo et al. (45), the analysis process consisted of three stages: preparation, organization, and reporting. Using units of meaning as the unit of analysis, the researchers repeatedly listened to the recordings, read the transcripts, and reviewed field notes to gain a comprehensive understanding of the data. Two researchers independently conducted open coding. The initial coding and category development were conducted inductively, grounded in the interview data. The coding process and preliminarily identified categories were regularly discussed and reviewed during team meetings, with the corresponding author, who has extensive experience in qualitative research, participating in these discussions. The coding process followed a stepwise abstraction process, progressing from initial codes to subcategories, categories, and finally main categories. The researchers first generated initial codes through open coding of the interview data, then continuously compared and grouped similar codes to form subcategories. These subcategories were further aggregated into categories and ultimately synthesized into main categories. All disagreements were resolved through collective discussion. Subsequently, the inductively derived categories were deductively organized and interpreted according to the micro-, meso-, and macro-levels of the Social Ecosystems Theory framework through team discussions until consensus was reached. The research findings were reported following the classification and abstraction of the open-coded data, with analysis results presented through descriptive text and representative quotations. All categories were derived from the data, and their consistency with the original materials was ensured through repeated verification.

### 2.5 Ethical considerations

This study received approval from the Ethics Review Committee of the data collection hospital (No.: 2025-189-K-156-02). All participants signed written informed consent, and their participation was voluntary.

### 2.6 Rigor

This study established rigor by ensuring principles of credibility, transferability, dependability, and confirmability (46). Researchers received systematic training in qualitative research, established relationships of trust with participants prior to interviews, and completed text transcriptions within 24 h of the interviews, thereby enhancing the study's credibility. Data were coded independently by two researchers, and discrepancies were resolved through team meetings to reach consensus. Analysis decisions were reflected upon and documented during regular team meetings, and the processes of data collection, analysis, and interpretation were continuously reviewed to ensure the dependability of the research process. The study employed purposive and maximum variation sampling to select diverse and representative participants, and the characteristics of the participants, the research context, and the study setting were clearly described, thereby ensuring transferability. Evaluation by an individual outside the research team was not performed in this study. At the same time, the researchers minimized the influence of their own subjective thinking through self-reflection. Throughout the research process, they maintained reflective journals, recording their reflections, assumptions, and potential biases after each interview and during key analytical stages such as coding and interpretation, and discussed these in team meetings. In addition, they conducted verification at the conclusion of each interview and promptly shared the analysis results with the participants, inviting them to review the transcripts to ensure confirmability. Throughout the writing of this paper, the study strictly adhered to the Comprehensive Standards for Reporting Qualitative Research (COREQ) (37) to ensure transparency and reproducibility.

## 3 Results

A total of 15 participants were ultimately included in this study and completed interviews, comprising eight male and seven female patients. Among them, four were aged 18–44 years, five were aged 45–59 years, and six were aged ≥60 years; one was illiterate, five had primary school education, six had junior high school education, one had senior high school education, and two had university education. Occupationally, three were farmers, four were workers, three were self-employed, two were company employees, and three were unemployed. Detailed participant information is presented in Table 2.

**Table 2:** General information of the interviewees (*n* = 15).

Variable	Category	*n*	%
Gender	Male	8	53.3%
	Female	7	46.7%
Age	18–44	4	26.7%
	45–59	5	33.3%
	≥60	6	40%
Occupation	Farmer	3	20%
	Worker	4	26.7%
	Self-employed	3	20%
	Staff	2	13.3%
	Unemployed	3	20%
Education	Illiteracy	1	6.7%
	Primary school	5	33.3%
	Junior high school	6	40%
	Senior high school	1	6.7%
	College graduate	2	13.3%
Cost category	Self pay	1	6.7%
	New rural cooperative insurance	12	80%
	Employee medical insurance	2	13.3%
Disease classification	Bacterial keratitis	2	13.3%
	Mycotic keratitis	8	53.3%
	Viral keratitis	4	26.7%
	Acanthamoeba keratitis	1	6.7%
The course of disease	< 3 months	9	60%
	≥3 months	6	40%

Data are presented as n (%).

As shown in Table 3, the code and main categories identified in the analysis were organized and interpreted using social ecosystem theory. At the microsystem level (Perceptions and Behavioral Adaptation Challenges Under Disease Impact), mesosystem level (Support and Tension in Patient Interactions with Family and Providers), and macrosystem level (challenges and needs in social network construction), facilitators and barriers influencing patient activation among individuals with infectious keratitis were identified.

**Table 3:** Mapping of main categories to the social ecosystems theory framework.

Level	Main categories	Categories
Microsystem	Perceptions and behavioral adaptation challenges under disease impact	Insufficient awareness and lack of care skills (B)
		Treatment burnout under symptom burden (B)
		Emotional burden amid disease uncertainty (B)
		Self-advocacy (F)
		Future-oriented positive coping (F)
		Redefining illness meaning (F)
Mesosystem	Support and tension in patient interactions with family and providers	Familial allyship in sustaining change (F)
		Support from health care providers (F)
		Peer support (F)
		Imbalance in family roles (B)
		Passive coping amidst communication barriers (B)
Macrosystem	Challenges and needs in building social networks	Social policy safeguards (F)
		Accessibility barriers to medical resources (B)
		Weak information support systems (B)
		Social alienation under public perception biases (B)

B, Barriers; F, Facilitators.

All findings directly from interview transcripts.

### 3.1 Microsystem level—perceptions and behavioral adaptation challenges under disease impact

At the micro level, the categories identified for facilitating patient activation were self-advocacy, future-oriented positive coping, and redefining illness meaning. Concurrently, insufficient awareness and lack of care skills, treatment burnout under symptom burden, and emotional burden amid disease uncertainty were recognized as barriers to activation for patients with infectious keratitis.

#### 3.1.1 Insufficient awareness and lack of care skills (barriers)

Interviews revealed widespread gaps in participants' understanding of the disease, particularly regarding its causes and progression. When eye infections manifested symptoms, most patients did not promptly seek medical treatment, instead opting for self-administered measures to alleviate discomfort. This approach often exacerbated disease progression, proving counterproductive.

Participants are identified by sequential codes (A1–A15) to protect their anonymity. The letter “A” denotes “Participant.”

One of the participants stated:

“When I was picking pumpkins, my eyes started itching. People in the village said I could wash them with herbal medicine. So I rinsed my eyes with it myself, but it didn't seem to help. Instead, my eyes started hurting even worse.” (A10)

Another participant stated:

“Even after my eyes started feeling uncomfortable, I kept working at the factory. To protect them, I wrapped alcohol-soaked tissues around my eyes. I thought alcohol could disinfect and prevent bacteria, but it just made the stinging worse.” (A1)

#### 3.1.2 Treatment burnout under symptom burden (barriers)

Some participants reported significant symptom burden from the disease, including eye pain, photophobia, tearing, and decreased vision. Due to the high frequency of medication required for treatment, some patients developed negative emotions and resistance. They feared the stinging pain during instillation, leading some to reduce their dosage without medical advice.

One of the participants stated:

“These eye drops are so painful when I put them in; I don't even want to use them. Sometimes I skip a few doses of this medication that's supposed to be administered every half hour.” (A2)

Additionally, participants reported that the impact of infectious keratitis extends beyond ocular symptoms. They often experience fatigue, poor appetite, and sleep disturbances. These symptoms affect patients' mental states and, to some extent, interfere with treatment adherence, representing a perceived barrier to patient activation.

One of the participants stated:

“I can't manage the eye drops with such frequent dosing schedules. During my hospital stay, I feel exhausted all day and just lie in bed. I often fall asleep and miss the scheduled drops.” (A7)

#### 3.1.3 Emotional burden amid disease uncertainty (barriers)

The varying degrees of vision loss and physical changes caused by the disease impose a significant psychological burden on patients. Faced with the uncertainty of disease progression and prognosis, patients express anxiety, uncertainty, and profound concerns about the future, which in turn undermine their confidence in disease management.

One of the participants stated:

I've been feeling very restless lately, under immense psychological pressure. My vision suddenly became blurry. How am I supposed to live like this? I'm just worried if my eyes will ever get better. (A1)

Another participant stated:

“I'm terribly worried I won't be able to live like a normal person anymore. I have to wear sunglasses everywhere I go. What's worse, my vision might never return to what it was before. But I'm still so young, I just can't accept this all at once.” (A11)

#### 3.1.4 Self-advocacy (facilitators)

Participants emphasized the importance of actively engaging in disease management and seeking support to achieve self-advocacy when facing illness challenges.

One of the participants stated:

“At first, I just let the doctors handle everything, and I never questioned the medications or treatment plans. But now I realize how crucial it is for us to be involved. I need to take responsibility for my own eyes, and I'll require self-care for a long time to come.” (A3)

#### 3.1.5 Future-oriented positive coping (facilitators)

Participants indicated that throughout their treatment journey, they needed to adjust their mindset to accept their condition with equanimity and respond proactively. By shifting their attitude toward the disease, they found meaning in persisting with treatment, thereby gaining inner freedom and strength.

One of the participants stated:

“I want to get my eyes better soon. I still want to dance with my friends in the square, and my daughter even said she'd take me traveling.” (A2)

Another participant stated:

“I believe that as long as I stick with the treatment, my eyes will gradually get better.” (A6)

#### 3.1.6 Redefining illness meaning (facilitators)

As treatment progressed, participants reported that their experience with the illness was not entirely negative. They received help and support from family and friends, feeling care and warmth from multiple sources. This helped them reinterpret their illness, giving it new meaning, and prompted them to reexamine their inner selves, ultimately changing their original outlook on life.

One of the participants stated:

“I‘ve gradually come to terms with the fact that illness is an inevitable part of everyone's life journey. Fortunately, during this time, I've received tremendous care and love from my family and friends.” (A3)

### 3.2 Mesosystem level—support and tension in patient interactions with family and providers

At the meso level, the core categories facilitating patient activation are familial allyship in sustaining change, support from health care providers and peer support. In contrast, passive coping amidst communication barriers and imbalance in family roles were identified as barriers to activation for patients with infectious keratitis.

#### 3.2.1 Familial allyship in sustaining change (facilitators)

Some participants reported significant support from family members for their active involvement in treatment and disease management. On one hand, this provided positive emotional value; on the other, it encouraged and reinforced healthy behaviors such as eye care routines, medication adherence, and regular follow-ups.

One of the participants stated:

“I often snapped at my family because of my poor vision, but they comforted and encouraged me. Feeling reassured helped me gradually improve and become more proactive in treatment.” (A4)

Another participant stated:

“I used to stay up late scrolling on my phone, but now my spouse keeps an eye on me and reminds me to rest my eyes.” (A15)

#### 3.2.2 Support from health care providers (facilitators)

Some participants indicated that the medical advice provided by healthcare providers contributed to lifestyle changes they made in managing their illness, while the empathetic approach also offered encouragement.

One of the participants stated:

“My doctor and nurses were very kind. They gave me a lot of advice, which was extremely helpful in learning how to care for my eyes. I can feel that they care for me and I have been respected by them, which is a good feeling.” (A11)

#### 3.2.3 Peer support (facilitators)

Participants mentioned that sharing their illnesses and experiences with fellow patients motivated each other and helped to reduce their anxiety about their illnesses, thus increasing their confidence in treatment.

One of the participants stated:

“I often chat with fellow inpatients about treatment insights. One patient had a much more severe case than mine but has recovered well, which also gives me great confidence.” (A8)

#### 3.2.4 Imbalance in family roles (barriers)

Participants felt guilty due to their dependence on caregivers for daily living and financial support. They believed their absence from family roles disrupted household functioning and increased the caregiver's burden, prompting a desire to quickly shed the “care recipient” identity. This sometimes led to thoughts of abandoning treatment or seeking early discharge to resume family and social roles, which may be related to the fact that Chinese culture is more inclined to consider the emotions and feelings of others.

One of the participants stated:

“My wife is disabled with lower body incontinence, waiting at home for me to return and care for her. My condition here won't improve anytime soon. Sometimes I feel it's better to go home sooner rather than later. I don't want to keep struggling with my eyes issue.” (A9)

Another participant stated:

“Our family is already struggling financially. After over twenty days of treatment here with little improvement, we've spent a fortune. My son works hard to earn money, and he's had to neglect his job to care for me here. I feel like I'm causing him immense trouble. I want to go home, I don't really want to continue treatment.” (A10)

#### 3.2.5 Passive coping amidst communication barriers (barriers)

Healthcare workers often face demanding schedules that limit their availability and opportunities for effective communication with patients. Conversely, patients may experience communication barriers due to limited health literacy or language differences. These challenges can significantly impede patients' comprehension of health-related information, hinder their active participation in disease-related discussions and decision-making processes, and ultimately diminish their patient activation.

One of the participants stated:

“The doctors here are too busy. I only see them during morning rounds, but the time is so short that they don't even have the chance to be able to say a few words.” (A7)

Another participant stated:

“I didn't get much schooling and don't speak Mandarin well, so I hardly talk to the doctors.” (A10)

### 3.3 Macrosystem level—challenges and needs in building social networks

At the macro level, the main category identified for facilitating patient activation was social policy safeguards. However, barriers to healthcare resource accessibility, weak information support systems, and social alienation under public perception bias were identified as barriers to patient activation in infectious keratitis.

#### 3.3.1 Social policy safeguards (facilitators)

Participants mentioned the support provided by the state and society. Medical insurance covered part of the costs for hospitalization and medications, offering treatment guarantees and alleviating their financial burdens to some extent.

One of the participants stated:

“The reimbursement rate from medical insurance is quite substantial, so I'm not overly concerned about the financial aspect. I feel fairly confident about receiving treatment here.” (A12)

Additionally, some participants expressed hopes for further improvements to medical insurance policies.

One of the participants stated:

“Transferring medical insurance across regions still requires certain procedures, and reimbursement rates differ from local standards. I hope this can be improved in the future.” (A10)

Another participant stated:

“I hope the coverage for these eye drops can be expanded in the future. Some imported eye drops I use are quite expensive and currently not covered.” (A6)

#### 3.3.2 Accessibility barriers to medical resources (barriers)

Due to the uneven distribution of medical resources across regions, renowned ophthalmology hospitals are concentrated in developed cities. Many primary healthcare facilities lack the capacity to promptly diagnose and treat infectious keratitis. Participants reported experiencing diagnostic and treatment delays before receiving a definitive diagnosis, leading to poorer disease outcomes. This uncertainty heightened their anxiety and diminished confidence in treatment.

One of the participants stated:

“When I first visited our local hospital, I didn't get a precise diagnosis. Some doctors said it was corneal edema; others said it was nothing serious, and everyone had different opinions. By the time I got here, the doctor said my eyes had already been neglected. I have no idea if my vision will recover.” (A4)

#### 3.3.3 Weak information support systems (barriers)

Participants indicated limited access to professional information and single-channel medical consultation, facing a shortage of effective information in disease management. Patients expressed a desire for ongoing follow-up after discharge to support better self-management, reduce recurrence, and improve prognosis.

One of the participants stated:

“We still lack sufficient understanding of this condition. I hope the ward can broadcast educational content about corneal diseases to provide patients with more information.” (A4)

Another participant stated:

“I've been worried about what to do when I get home. I hope there will be a way for me to seek advice after I'm discharged.” (A3)

#### 3.3.4 Social alienation under public perception biases (barriers)

Within the Chinese cultural context, people generally prefer to provide value to others rather than cause trouble, so most are reluctant to disclose their illness to family and friends, even refusing to seek support. This leads patients to develop social withdrawal and isolation, resulting in decreased activation.

One of the participants stated:

“I haven't told any relatives or friends because I don't see this as a good thing. If I can handle it myself, I try not to tell them. I don't want them to worry, and it's inconvenient for them to travel all this way to visit me.” (A13)

As the disease progresses with a poor prognosis, some patients experience changes in ocular appearance, including corneal opacity and scarring. This often leads to varying degrees of discrimination, significantly hindering their participation in social activities and exacerbating social isolation.

One of the participants stated:

“I'm currently on a temporary leave of absence from school because of my eyes. I've been bullied at school. My classmates always stare at my eyes, treating me like some kind of freak. Some even wonder if it's contagious.” (A8)

Another participant stated:

“Over the past two years, I've spent most of my time at home due to recurring eye infections. I'm terrified that if people saw my eyes like this, they'd all run away. (sigh)” (A5)

## 4 Discussion

Based on social ecosystem theory, this study reveals that patient activation in individuals with infectious keratitis is not determined by a single factor but rather by the interaction of multiple factors across micro, meso, and macro systems. This indicates that patient activation in this population is influenced by the complex interplay of individual, interpersonal, and societal factors.

This study found that at the micro level, patients face adaptive challenges in disease cognition and behavioral management. Some patients reported a lack of disease knowledge and self-care skills, which hindered their timely access to medical care and disease management; a study by Puri et al. also expressed similar findings (47). Due to low educational levels and insufficient financial resources, these patients have low healthcare awareness, which is consistent with the results of previous studies (14, 48). Our results also indicate that some patients failed to seek timely medical care and instead tended to resort to self-administered relief measures, which is consistent with findings in the existing literature (49). It is worth noting that, unlike the study by Li et al., which focused on older adult(s) cancer patients (35), this study observed across a broader age range that a lack of information evaluation skills persists even among young patients with high levels of education and awareness of healthy behaviors. From the perspective of the social ecosystems theory, this phenomenon cannot be attributed solely to individual capability deficits but is deeply influenced by meso- and macro-level factors (34). The underlying reasons may be related to patients' difficulty in obtaining authoritative guidance through professional channels such as doctor-patient communication, as well as the weakness of their social information support systems. To address the diverse needs of these patients, a tiered educational strategy can be implemented. Healthcare providers can analyze patient profiles to systematically assess the educational barriers and personal preferences each patient faces and develop personalized health education strategies based on these findings, thereby expanding and enriching patients' channels for acquiring knowledge (50). Based on the findings of this study, we recommend that healthcare providers offer basic educational materials in local dialects and with illustrations to patients with lower levels of education, and use traditional methods such as lectures, informational posters, and hands-on training to provide disease education. Concurrently, video tutorials (51) can be used to provide guidance on specific care skills, such as administering eye drops and performing eye care, combined with hands-on training to help patients and their caregivers master these skills. For patients with higher educational attainment, interactive e-learning modules can be introduced. Through case studies and online quizzes, these modules help patients establish a framework for evaluating information while enabling continuous educational follow-up and optimization (13). A previous study has shown that misleading or inaccurate information may lead to inappropriate treatment or delayed care (52). Therefore, we recommend that, regardless of patient type, medically reviewed public health education content be disseminated through digital channels such as WeChat and mini-programs to ensure the accuracy and reliability of the information.

According to the social ecosystems theory, individuals' psychological experiences and behaviors continuously and dynamically interact with other systems (34), and the findings of the present study corroborate this view. At the micro level, we observed that patients' distress over symptoms and emotional burden negatively impacted disease treatment and management; meanwhile, support from family members, peers, and healthcare providers at the meso level served as a key facilitating factor, effectively alleviating patients' negative emotions and psychological stress, consistent with previous research (53). Similarly, Li et al. also found that emotional support from family members promotes patient activation among older adult(s) cancer patients (35). It is worth noting that participants in this study expressed feelings of guilt for being a burden to their families due to their illness, as well as a tendency to reduce their use of medical services to avoid adding to the family' s burden. From a cultural analysis perspective, influenced by China's collectivist culture, patients receiving care from their families feel guilty about being perceived as a burden on the family (54). This may lead them to adopt avoidant coping behaviors, which in turn makes family role imbalance a significant barrier at the meso level. In collectivist cultures, there is a high degree of interdependence between individuals and members of their ingroup (such as the family), and group goals are often prioritized over individual needs (55). Previous research has also shown that patients bearing heavier family responsibilities tend to be less proactive in seeking medical care (56). This reveals how macro-level cultural values exert a profound influence on individual health behaviors through meso-level family interaction patterns. At the macro level, the findings of the present study showed that patients lack social understanding and support and face social alienation resulting from social cognitive biases. Some patients mentioned in interviews that they “didn't want to worry their loved ones” or “didn't want to be a burden to others”; these statements reflect their restraint in expressing their own needs and emotions. This phenomenon may be understood from the perspective of Confucian cultural values. In traditional Chinese Confucian thought, “Ren” refers to inner moral emotions, while “Li” denotes external behavioral norms, emphasizing restraint in emotional expression (57). Against this cultural backdrop, patients often tend to suppress their feelings and avoid seeking support from others (58). At the same time, misunderstandings and prejudices surrounding infectious eye diseases further exacerbate patients' sense of isolation. Unlike common chronic diseases, infectious keratitis—with its visible symptoms (corneal opacity and visual impairment)—is subject to the dual impact of the stigma associated with infectious diseases: patients not only refrain from speaking out due to cultural norms but also actively withdraw because of the public's misunderstandings about contagion. This social isolation, in turn, undermines patients' willingness to participate in social activities and manage their condition. Therefore, we recommend that family education should not only emphasize the importance of support but also guide family members on how to provide assistance in ways that alleviate the patient's psychological burden. For example, understanding patients' genuine needs through open communication, rather than offering one-sided support, helps alleviate their psychological stress (59). Secondly, the findings of the present study showed that healthcare professionals should assist patients in integrating social resources to build a multi-party support system involving hospitals, families, and communities. They should encourage patients to actively express their feelings and guide family members and friends to provide ongoing emotional support and practical assistance. Furthermore, peer support groups can be established to connect patients with others who have had similar experiences, enabling them to jointly access disease-related information, treatment recommendations, and coping strategies, as well as seek external assistance, thereby gaining social support to alleviate internal family pressures and enhance patient motivation (60). At the same time, it is recommended to correct misinformation through community public health education strategies (61) to reduce misunderstandings and discrimination regarding infectious keratitis and support patients' reintegration into society (62).

At the meso level, we found that a lack of effective doctor-patient communication is a major barrier. Research indicates that China's current healthcare landscape is characterized by a notable disparity between the number of patients and healthcare providers, thereby limiting the time available for doctor-patient communication (63). Communication barriers between patients and healthcare providers reduce patients' access to health information, subsequently lowering patient activation—a finding also reflected in other studies (64). This study also found that patients generally adopt a passive communication with ealthcare providers. This may be related to China's culture of high power distance. In this cultural context, medical authority is highly respected, and doctor-patient communication is often one-sided, with patients rarely questioning or challenging doctors' decisions (65). Consequently, Chinese patients tend to follow a provider-centered communication model, with limited active participation in their own care (66). Research by Saeed et al. found that a positive doctor-patient relationship can enhance patients' sense of trust and engagement, which is crucial for improving patients' passive state (67). Our findings revealed that patients had clear expectations of healthcare providers, including receiving clear and understandable disease information and treatment guidance, being respected and listened to during communication, and having adequate attention and sufficient time for interaction. This suggests that healthcare providers should adopt patient-centered communication strategies tailored to patients' psychological characteristics (68). They should actively guide patients to participate in disease management, jointly develop treatment and rehabilitation plans with patients, and demonstrate empathy, respect, and an inclusive attitude (69). At the same time, structured tools such as question lists can be provided to patients to help them organize key issues and make more efficient use of communication time (70). These strategies may help facilitate a shift from passive acceptance of care to active maintenance of healthy behaviors.

At the macro level, our findings indicate that China's current social policies serve as primary facilitators, providing patients with certain treatment guarantees and alleviating financial burdens. However, current policies still have certain limitations and require further refinement in the future. At the same time, barriers to healthcare access and weak information support systems constitute major obstacles. Due to the uneven distribution of medical resources across regions (71), there remains a gap between the diagnostic and treatment capabilities of primary healthcare institutions in China and the requirements for high-quality primary care services. It is worth noting that, unlike most chronic diseases, vision loss associated with infectious keratitis is urgent and carries the risk of irreversibility. Patients must receive specialized ophthalmic care within a limited time window; this time-sensitivity makes the consequences of delayed care even more severe. Delayed treatment exacerbates the progression of infectious keratitis, leading to poor clinical outcomes and, consequently, a reduced quality of life (72, 73). Furthermore, patients may require long-term follow-up and monitoring after discharge, yet they face the dilemma of inadequate support systems, including difficulties in accessing medical information, long distances to healthcare facilities, and transportation challenges (74). Therefore, it is necessary to further strengthen the development of primary ophthalmic healthcare services. It is recommended to improve the allocation of resources between urban and rural areas, enhance medical service capacity, increase the accessibility of ophthalmic nursing services, strengthen referral networks, and encourage early and appropriate treatment (71) to reduce delays and ultimately improve clinical outcomes. In parallel, future research could further explore the potential application of artificial intelligence and digital health technologies in ophthalmology. For instance, AI-driven telemedicine platforms may offer early assessment and diagnostic support for resource-limited areas (75–77), while electronic medical record-based intelligent follow-up systems could help patients better manage their condition through medication reminders, visit notifications, and home care guidance (78). These approaches require further research to validate their feasibility and effectiveness.

## 5 Strengths and limitations

This study employed qualitative methods to explore in depth the authentic experiences of patients with infectious keratitis regarding disease management and health behaviors. It identified key factors that may facilitate or hinder patient activation across micro, meso, and macro levels, providing valuable empirical evidence for subsequent interventions. However, this study has several limitations. First, the sample was drawn from only one hospital; while this facilitated in-depth analysis within a specific healthcare context, the findings may be influenced by regional differences and the sociocultural context of China (such as collectivist values and a high-power-distance culture), thereby limiting their direct generalizability to other sociocultural contexts. Future research could validate the findings of this study across different cultural contexts and further explore cross-cultural differences in patient activation. Second, interviews were conducted within a single timeframe without long-term patient follow-up, limiting the ability to capture changes in patient activation and its influencing factors over time. Third, while qualitative methods provided valuable insights into patient experiences, the study did not include quantitative data to assess the specific impact of various factors, nor did it incorporate other eye disease patient groups for comparison. Future research could adopt a comparative design or combine quantitative research methods to further validate the findings of this study. Additionally, this study focused solely on the patient perspective, excluding healthcare providers and caregivers. Future research integrating multiple perspectives could further develop a comprehensive understanding framework.

## 6 Conclusion

In summary, drawing on the social ecosystems theory, this study found that although some patients with infectious keratitis are able to mobilize individual and relational resources to support their activation, their ability to actively manage their health remains constrained by multiple micro-, meso-, and macro-level factors. Based on these findings, three key insights directly relevant to clinical practice have been identified. First, the cognitive gap at the micro level indicates an urgent need to establish a tiered, highly accessible patient education system. Second, communication barriers between physicians and patients and imbalanced family roles at the meso level suggest that patient-centered communication strategies should be implemented, and a multi-stakeholder support network involving hospitals, families, and communities should be established. Third, the lack of access to medical resources at the macro level and the weakness of information support systems highlight the urgent need to strengthen primary-care ophthalmology capacity. Future research could further explore the potential applications of artificial intelligence and digital health technologies in the field of ophthalmology. These findings underscore the importance of supporting patients' transition from passive treatment to active participation, which is crucial for improving the timeliness of diagnosis, treatment adherence, and long-term prognosis among patients with infectious keratitis.

## Data Availability

The raw data supporting the conclusions of this article will be made available by the authors, without undue reservation.
