# Supplementary material for: Analysis of barriers and facilitators to patient activation among patients with infectious keratitis from a social ecosystems theory perspective: a qualitative study in the Chinese context
Source: Front Public Health. 2026 Oct 7;14:1881288. doi: 10.3389/fpubh.2026.1881288 (PMC13683699; doi:10.3389/fpubh.2026.1881288)
Supplement: Supplementary file 1 [file Supplementary_File_1.docx]

**Coding tree: progression from initial codes to main categories**

| **Representative quote** | **Initial codes** | **Subcategories** | **Categories** | **Main Categories** | **Level** |
| --- | --- | --- | --- | --- | --- |
| “When I was picking pumpkins, my eyes started itching. People in the village said I could wash them with herbal medicine. So I rinsed my eyes with it myself, but it didn't seem to help. Instead, my eyes started hurting even worse.”(A10) | Followed a folk remedy and rinsed the eyes with herbal remedies | Lack of awareness of the disease | Insufficient Awareness and Lack of Care Skills（B） | Perceptions and Behavioral Adaptation Challenges Under Disease Impact | Microsystem |
| “Even after my eyes started feeling uncomfortable, I kept working at the factory. To protect them, I wrapped alcohol-soaked tissues around my eyes. I thought alcohol could disinfect and prevent bacteria, but it just made the stinging worse.” (A1) | Spray alcohol on a tissue to protect eyes | Incorrect eye care methods |  |  |  |
| “These eye drops are so painful when I put them in, I don't even want to use them. Sometimes I skip a few doses of this medication that's supposed to be administered every half hour.” (A2) | Missed doses due to discomfort caused by the eye drops | Treatment-related symptom burden | Treatment Burnout Under Symptom Burden（B） |  |  |
| “I can't manage the eye drops with such frequent dosing schedules. During my hospital stay, I feel exhausted all day and just lie in bed. I often fall asleep and miss the scheduled drops.” (A7) | Difficulty adhering to high-frequency medication due to fatigue | Treatment-related discomfort and fatigue |  |  |  |
| “I've been feeling very restless lately, under immense psychological pressure. My vision suddenly became blurry. How am I supposed to live like this? I'm just worried if my eyes will ever get better.” (A1) | The uncertainty surrounding vision recovery causes anxiety and stress | Anxiety and uncertainty about prognosis | Emotional Burden Amid Disease Uncertainty（B） |  |  |
| “I'm terribly worried I won't be able to live like a normal person anymore. I have to wear sunglasses everywhere I go. What's worse, my vision might never return to what it was before. But I'm still so young, I just can't accept this all at once.” (A11) | Worry that vision loss will affect normal life | Worry and uncertaintyabout prognosis |  |  |  |
| “At first, I just let the doctors handle everything, and I never questioned the medications or treatment plans. But now I realize how crucial it is for us to be involved. I need to take responsibility for my own eyes, and I'll require self-care for a long time to come.” (A3) | Shifting from passivedependence to active participation and recognizing the importance of participating in disease management | Awareness of self-responsibility in disease management | Self-Advocacy（F） |  |  |
| “I want to get my eyes better soon. I still want to dance with my friends in the square, and my daughter even said she'd take me traveling.” (A2) | Longing to dance with friends and travel with family after eye recovery | Expectation of future life | Future-Oriented Positive Coping（F） |  |  |
| “I believe that as long as I stick with the treatment, my eyes will gradually get better.” (A6) | Belief that treatment adherence will lead to vision recovery | Belief in and persistence with recovery |  |  |  |
| “I've gradually come to terms with the fact that illness is an inevitable part of everyone's life journey. Fortunately, during this time, I've received tremendous care and love from my family and friends. ” (A3) | Realizing that accepting illness is a part of life and feeling the care and love from family and friends | Illness acceptance and meaning reconstruction | Redefining Illness Meaning（F） |  |  |
| “I often snapped at my family because of my poor vision, but they comforted and encouraged me. Feeling reassured helped me gradually improve and become more proactive in treatment.” (A4) | Emotional distress due to poor vision, alleviated by family comfort and encouragement | Comfort and encouragement from family | Familial Allyship in Sustaining Change（F） | Support and Tension in Patient Interactions with Family and Providers | Mesosystem |
| “I used to stay up late scrolling on my phone, but now my spouse keeps an eye on me and reminds me to rest my eyes.” (A15) | Improved eye care habits under spouse's supervision | Family supervision and behavioral correction |  |  |  |
| “My doctor and nurses were very kind. They gave me a lot of advice, which was extremely helpful in learning how to care for my eyes. I can feel that they care for me and I have in being respected by them, which is a good feeling.” (A11) | Healthcare providers offered professional advice while demonstrating respect and care | Professional guidance and emotional support from healthcare providers | Support from Health Care Providers（F） |  |  |
| “I often chat with fellow inpatients about treatment insights. One patient had a much more severe case than mine but has recovered well, which also gives me great confidence.” (A8) | Sharing treatment experiences with other patients and gaining confidence from others' recovery journeys | Peer experience sharing and emotional support | Peer Support（F） |  |  |
| “My wife is disabled with lower body incontinence, waiting at home for me to return and care for her. My condition here won't improve anytime soon. Sometimes I feel it's better to go home sooner rather than later. I don't want to keep struggling with my eyes issue.” (A9) | Desire to abandon treatment to return home and care for disabled wife | Conflict between family care responsibilities | Imbalance in Family Roles（B） |  |  |
| “Our family is already struggling financially. After over twenty days of treatment here with little improvement, we've spent a fortune. My son works hard to earn money, and he's had to neglect his job to care for me here. I feel like I'm causing him immense trouble. I want to go home, I don't really want to continue treatment.” (A10) | Feeling like a burden to son and wanting to give up treatment | Feeling of being a burden to family |  |  |  |
| “The doctors here are too busy. I only see them during morning rounds, but the time is so short that they don't even have the chance to be able to say a few words.” (A7) | The doctors are too busy to have a chance to talk with patients. | Insufficient time for patient-provider communication | Passive Coping Amidst Communication Barriers（B） |  |  |
| “I didn't get much schooling and don't speak Mandarin well, so I hardly talk to the doctors.” (A10) | A low level of education and language barriers hinder communication with doctors | Language and cultural barriers |  |  |  |
| “The reimbursement rate from medical insurance is quite substantial, so I'm not overly concerned about the financial aspect. I feel fairly confident about receiving treatment here.” (A12) | Health insurance reimbursement provides a certain level of coverage for medical treatment | Medical insurance coverage | Social Policy Safeguards（F） | Challenges and Needs in Building Social Networks | Macrosystem |
| “Transferring medical insurance across regions still requires certain procedures, and reimbursement rates differ from local standards. I hope this can be improved in the future.” (A10) | Expectations for Improvements to Out-of-Area Health Insurance Procedures and Reimbursement Rates | Medical insurance policy needs improvement |  |  |  |
| “I hope the coverage for these eye drops can be expanded in the future. Some imported eye drops I use are quite expensive and currently not covered.” (A6) | Hope that medication coverage under medical insurance will be expanded | Need to expand drug reimbursement coverage |  |  |  |
| "When I first visited our local hospital, I didn't get a precise diagnosis. Some doctors said it was corneal edema, others said it was nothing serious, everyone had different opinions. By the time I got here, the doctor said my eyes had already been neglected. I have no idea if my vision will recover." (A4) | Misdiagnosis at a primary care hospital led to a delay in treatment | Limited primary care capacity | Accessibility Barriers to Medical Resources（B） |  |  |
| “We still lack sufficient understanding of this condition. I hope the ward can broadcast educational content about corneal diseases to provide patients with more information.” (A4) | Expectation of disease education programs offered by the hospital | Need for in-hospital health education | Weak Information Support Systems（B） |  |  |
| “I’ve been worried about what to do when I get home. I hope there will be a way for me to seek advice after I’m discharged.” (A3) | Worry about the lack of consultation channels for post-discharge home care | Lack of post-discharge information support |  |  |  |
| “I haven't told any relatives or friends because I don't see this as a good thing. If I can handle it myself, I try not to tell them. I don't want them to worry, and it's inconvenient for them to travel all this way to visit me.” (A13) | Reluctance to inform relatives or friends due to unwillingness to burden others | Self-isolation and avoidance of seeking help | Social Alienation Under Public Perception Biases（B） |  |  |
| “I'm currently on a temporary leave of absence from school because of my eyes. I've been bullied at school. My classmates always stare at my eyes, treating me like some kind of freak. Some even wonder if it's contagious.” (A8) | Experiencing discrimination due to eye appearance and its impact on academic progress | School discrimination and exclusion |  |  |  |
| “Over the past two years, I've spent most of my time at home due to recurring eye infections. I'm terrified that if people saw my eyes like this, they'd all run away. (sigh)” (A5) | Staying at home for a long time without socializing due to repeated infections | Decreased social participation |  |  |  |

Note: This table presents the analytical progression from initial codes to subcategories, categories, and main themes. It is intended to enhance analytical transparency and is not a results table.
