# Supplementary material for: Analysis of barriers and facilitators to patient activation among patients with infectious keratitis from a social ecosystems theory perspective: a qualitative study in the Chinese context
Source: Front Public Health. 2026 Oct 7;14:1881288. doi: 10.3389/fpubh.2026.1881288 (PMC13683699; doi:10.3389/fpubh.2026.1881288)
Supplement: Supplementary file 2 [file Supplementary_File_2.docx]

**COREQ (COnsolidated criteria for REporting Qualitative research) Checklist**

A checklist of items that should be included in reports of qualitative research. You must report the page number in your manuscript where you consider each of the items listed in this checklist. If you have not included this information, either revise your manuscript accordingly before submitting or note N/A.

| **Topic** | **Item No.** | **Guide Questions/Description** | **Reported on Page No.** |
| --- | --- | --- | --- |
| **Domain 1: Research team and reflexivity** |  |  |  |
| *Personal characteristics* |  |  |  |
| Interviewer/facilitator | 1 | Which author/s conducted the interview or focus group? | Page9 , Methods – Data Collection |
| Credentials | 2 | What were the researcher’s credentials? E.g. PhD, MD | Page9 , Methods – Data Collection |
| Occupation | 3 | What was their occupation at the time of the study? | Page9 , Methods – Data Collection |
| Gender | 4 | Was the researcher male or female? | Page9 , Methods – Data Collection |
| Experience and training | 5 | What experience or training did the researcher have? | Page9 , Methods – Data Collection |
| *Relationship with participants* |  |  |  |
| Relationship established | 6 | Was a relationship established prior to study commencement? | Page10 , Methods – Data Collection |
| Participant knowledge of the interviewer | 7 | What did the participants know about the researcher? e.g. personal goals, reasons for doing the research | Page9-10 , Methods – Data Collection |
| Interviewer characteristics | 8 | What characteristics were reported about the inter viewer/facilitator? e.g. Bias, assumptions, reasons and interests in the research topic | Page9-10 , Methods – Data Collection |
| **Domain 2: Study design** |  |  |  |
| *Theoretical framework* |  |  |  |
| Methodological orientation and Theory | 9 | What methodological orientation was stated to underpin the study? e.g.  grounded theory, discourse analysis, ethnography, phenomenology, content analysis | Page 7, Methods – Research Design |
| *Participant selection* |  |  |  |
| Sampling | 10 | How were participants selected? e.g. purposive, convenience, consecutive, snowball | Page 8, Methods – Participants and Sampling |
| Method of approach | 11 | How were participants approached? e.g. face-to-face, telephone, mail, email | Page9 , Methods – Data Collection |
| Sample size | 12 | How many participants were in the study? | Page 8, Methods – Participants and Sampling |
| Non-participation | 13 | How many people refused to participate or dropped out? Reasons? | Page 8, Methods – Participants and Sampling |
| *Setting* |  |  |  |
| Setting of data collection | 14 | Where was the data collected? e.g. home, clinic, workplace | Page10 , Methods – Data Collection |
| Presence of nonparticipants | 15 | Was anyone else present besides the participants and researchers? | Page10 ,Methods – Data Collection |
| Description of sample | 16 | What are the important characteristics of the sample? e.g. demographic data, date | Page10 ,Result |
| *Data collection* |  |  |  |
| Interview guide | 17 | Were questions, prompts, guides provided by the authors? Was it pilot tested? | Page10 ,Methods – Data Collection |
| Repeat interviews | 18 | Were repeat inter views carried out? If yes, how many? | Page10 ,Methods – Data Collection |
| Audio/visual recording | 19 | Did the research use audio or visual recording to collect the data? | Page11 ,Methods – Data Collection |
| Field notes | 20 | Were field notes made during and/or after the inter view or focus group? | Page10 ,Methods – Data Collection |
| Duration | 21 | What was the duration of the inter views or focus group? | Page10 ,Methods – Data Collection |
| Data saturation | 22 | Was data saturation discussed? | Page 8, Methods – Participants and Sampling |
| Transcripts returned | 23 | Were transcripts returned to participants for comment and/or correction? | Page11 , Methods – Data Collection |
| **Domain 3: analysis and findings** |  |  |  |
| *Data analysis* |  |  |  |
| Number of data coders | 24 | How many data coders coded the data? | Page 12-13, Methods – Data Analysis |
| Description of the coding tree | 25 | Did authors provide a description of the coding tree? | Page 12-13, Methods – Data Analysis |
| Derivation of themes | 26 | Were themes identified in advance or derived from the data? | Page 12-13, Methods – Data Analysis |
| Software | 27 | What software, if applicable, was used to manage the data? | Page 12,Methods – Data Analysis |
| Participant checking | 28 | Did participants provide feedback on the findings? | Page11 , Methods – Data Collection |
| *Reporting* |  |  |  |
| Quotations presented | 29 | Were participant quotations presented to illustrate the themes/findings?  Was each quotation identified? e.g. participant number | Page 15-26, Results |
| Data and findings consistent | 30 | Was there consistency between the data presented and the findings? | Page 15-26, Results |
| Clarity of major themes | 31 | Were major themes clearly presented in the findings? | Page 26-31, Discussion |
| Clarity of minor themes | 32 | Is there a description of diverse cases or discussion of minor themes? | Page 26-31, Discussion |

Developed from: Tong A, Sainsbury P, Craig J. Consolidated criteria for reporting qualitative research (COREQ): a 32-item checklist for interviews and focus groups. *International Journal for Quality in Health Care*. 2007. Volume 19, Number 6: pp. 349 – 357

**Once you have completed this checklist, please save a copy and upload it as part of your submission. DO NOT** **include this checklist as part of the main manuscript document. It must be uploaded as a separate file.**
